# Korean translation of the CONSORT 2010 Statement: updated guidelines for reporting parallel group randomized trials

**DOI:** 10.4178/epih/e2014029

**Published:** 2014-11-08

**Authors:** Jun Suh Lee, Soyeon Ahn, Kyoung Ho Lee, Jee Hyun Kim

**Affiliations:** 1 Department of Surgery, Seoul St. Mary’s Hospital, College of Medicine, Catholic University of Korea, Seoul, Korea; 2 Division of Statistics, Medical Research Collaborating Center, Seoul National University Bundang Hospital, Seongnam, Korea; 3 Department of Radiology, Seoul National University College of Medicine, Seoul National University Bundang Hospital, Seongnam, Korea; 4 Department of Internal Medicine, Seoul National University College of Medicine, Seoul National University Bundang Hospital, Seongnam, Korea

**Keywords:** Randomized controlled trials, Research design, Guideline

## Abstract

The Consolidated Standards of Reporting Trials (CONSORT) 2010 Statement, updated in March 2010, includes a 25-item checklist and flow diagram. Adherence to this statement is a minimum requirement for the complete, clear, and transparent reporting of randomized trials. We translated the CONSORT 2010 Statement into Korean to promote the widespread adherence to CONSORT in South Korea and to facilitate the adoption of complete, clear, and transparent reporting. The Korean version of the CONSORT is available at http://www.e-epih.org/.

## THE CONSORT 2010 STATEMENT

The Consolidated Standards of Reporting Trials (CONSORT) statement is a reporting guideline for randomized controlled trials (RCTs). It is expected that the use of CONSORT facilitates high quality reporting and therefore improves the assessment of RCTs [1-3].

The CONSORT 2010 Statement, updated in March 2010, includes a 25-item checklist and flow diagram, which are minimum requirements that should be adhered to for the complete, clear, and transparent reporting of RCTs. We translated the CONSORT 2010 Statement into Korean to promote the widespread use of the CONSORT statement in South Korea.

## THE TRANSLATION PROCESS

Between June 2012 and February 2013, the CONSORT 2010 Statement was translated into Korean. Volunteers who understand the value of the CONSORT 2010 Statement and the need of its introduction to Korean readers performed the translation. We obtained permission from the CONSORT 2010 group for Korean translation, and the translation process was done according to the guidelines provided by the CONSORT 2010 group. One translator (Ahn S) proceeded with the forward translation of the CONSORT 2010 Statement [4]. Another bilingual (Korean-English) translator (Lee JS), who was not involved in the forward translation, performed the backward translation. The penultimate version of translation was sent to the CONSORT group for review, and some discrepancies were noted in the backward translation. The translators modified these discrepancies as well as several other ambiguous expressions, and then two independent reviewers (Lee KH and Kim JH) reviewed these discrepancies. All translators and reviewers were born in South Korea, had lived in an English-speaking country for at least 1 year prior to translation, and are fluent in both Korean and English.

## IMPLICATIONS

We believe that the translation of the CONSORT 2010 Statement will facilitate the widespread adoption of the CONSORT statement in South Korea and the clear, complete, and transparent reporting of future RCTs. For trialists, adherence to the CONSORT statement helps to not only report but also design and conduct trials. For reviewers and readers, the completeness of reported trials supports the critical appraisal and interpretation of these data, and aids in the synthesis of evidence subject to systematic review. For participants, a translated version of the terminology and process may help to broaden their understanding of these trials. The Korean version of the CONOSRT 2010 Statement is available at http://www.e-epih.org/ as a supplemental material.
