# Supplementary material for: Korean translation of the CONSORT 2010 Statement: updated guidelines for reporting parallel group randomized trials
Source: Epidemiol Health. 2014 Nov 8;36:e2014029. doi: 10.4178/epih/e2014029 (PMC4371386; doi:10.4178/epih/e2014029)
Supplement: Supplementary file 1 [file epih-36-e2014029-supplementary.pdf]

SUPPLEMENTAL MATERIAL

■ SUPPLEMENTARY FILE

Korean translation of the CONSORT 2010 Statement: updated guidelines for reporting parallel group randomized trials

Jun Suh Lee[1], Soyeon Ahn[2], Kyoung Ho Lee[3], Jee Hyun Kim[4]

[1]Department of Surgery, Seoul St. Mary's Hospital, College of Medicine, Catholic University of Korea, Seoul; [2]Division of Statistics, Medical Research Collaborating Center, Seoul National University Bundang Hospital, Seongnam; [3]Department of Radiology, Seoul National University College of Medicine, Seoul National University Bundang Hospital, Seongnam; [4]Department of Internal Medicine, Seoul National University College of Medicine, Seoul National University Bundang Hospital, Seongnam, Korea

CONSORT 2010 한글 번역의 목적

무작위 임상시험은 근거바탕의학의 과학적 방법론의 핵심이며, 임상 진료와 의료 정책과 같은 중요한 의학적 결정권의 근거가 된다. 그러나 특히 무작위 임상시험의 경우, 적절하게 설계되고 수행되고 보고된 경우라야 개입의 효과를 객관적으로 평가할 수 있다. CONSORT (Consolidated Standards of Reporting Trials)는 무작위 임상시험 보고의 질을 향상시키기 위한 목적으로 개발되었으며, 시험 설계와 수행 과정을 투명하게 보고하도록 이끌어주는 보고 지침이다. 본 고에서 CONSORT 2010의 공식 한글번역본 및 번역과정을 소개하고자 한다.

대상 및 방법

CONSORT 2010은 25개 항목으로 구성된 점검표, 흐름도, 그리고 CONSORT 2010 지침으로 구성되어 있다. CONSORT 2010의 한글번역은, 한글번역의 필요성을 제기하고 자발적으로 번역에 참여한 번역자에 의해 진행되었다. 한글번역의 대상은 점검표와 흐름도를 포함한 CONSORT 2010 지침이며 Annals of Internal Medicine에 발표된 판본을 참고하였다 [1]. CONSORT 그룹에 의뢰하여 번역에 대한 승인을 얻은 후, 사전번역 및 사후번역 및 CONSORT 그룹의 분석 과정, 재검토 과정을 거쳤다.

사전번역 및 사후번역

한국어 및 영어에 능통한 2인의 번역자는 CONSORT 그룹으로부터 한글번역에 대한 승인을 얻었다. 1명의 번역자가 한국어로 CONSORT 2010 사전번역(forward translation)을 수행하였고, 이 한글 사전번역본을 바탕으로 독립적인 1명의 번역자가 사후번역(back translation)을 수행하였다.

재검토 및 최종본

CONSORT 그룹은 사후번역본을 원본과 대비하여 수정이 필요한 항목을 분석하였다. CONSORT 그룹의 분석사항을 토대로, 번역에 참여하지 않은 임상시험 전문가인 2명의 검토자가 미묘한 용어 및 어색한 표현을 수정하였다. 이런 재검토 과정을 수회 거쳐 번역자는 CONSORT 2010 한글번역 최종본을 완성하였다.

결과

점검표와 흐름도를 포함한 CONSORT 2010 지침 한글번역본이다 (Supplementary Table 1, Supplementary Figure 1).

고찰

CONSORT 2010의 출판 연도를 고려할 시, 한글번역본 출판은 늦은 편이다. 그러나 국내에서 임상시험을 CONSORT 지침에 따라 보고하는 것이 체계화되지 않았고, 심지어 보고 지침의 존재를 모르는 경우도 있다. CONSORT 2010 한글번역본은 국내 무작위 임상시험의 투명한 보고에 기여할 것으로 기대한다. 또한 CONSORT 2010 한글번역본에서 소개된 임상시험 용어와 절차는, 임상시험 전문가뿐만 아니라 다양한 배경으로 구성된 연구대상자 및 공동연구자에게 임상시험에 대한 이해를 넓힐 것으로 기대된다. 연구자가 CONSORT 2010 지침을 충실히 이행할 경우 완전하고 명료하고 투명한 보고가 가능하며 앞으로의 시험수행의 설계와 수행에 도움이 될 것이다.

참고문헌

1. Schulz KF, Altman DG, Moher D, Group C. CONSORT 2010 statement: updated guidelines for reporting parallel group randomized trials. Ann Intern Med 2010;152:726-732.

CONSORT 2010 지침: 평행설계 무작위 임상시험을 보고하기 위해 개정된 지침

Kenneth F. Schulz PhD, MBA; Douglas G Altman, DSc; and David Moher, PhD for the CONSORT Group

초록

CONSORT (Consolidated Standards of Reporting Trials) 지침은 무작위 임상시험 보고의 질을 향상시키기 위해 전세계적으로 사용된다. Schulz와 동료들은 새로운 방법론과 누적된 경험을 바탕으로 개정된 보고 지침인 CONSORT 지침의 최신판으로, CONSORT 2010을 기술한다.

서론

무작위 임상시험은 적절하게 설계되고 수행되고 보고된 경우에만 보건의료 개입을 평가하는 최선의 방법이 된다. 그러나, 만약 방법론적인 엄밀함을 따르지 않는다면, 무작위 임상시험은 치우친 결과를 초래할 수 있다 [1]. 시험을 정확하게 평가하기 위해서는 출판된 보고를 읽는 독자들은 방법론과 발견에 대한 완전하고 명료하고 투명한 정보를 필요로 한다. 안타깝게도, 많은 시험의 저자들이 이런 결정적인 정보에 대한 명쾌하고 완결된 설명을 소홀히 하기 때문에, 시도된 평가가 종종 수포로 돌아간다 [2-4].

이런 불충분한 보고가 1996년 CONSORT (Consolidated Standards of Reporting Trials) 지침 원본 개발 [5] 및 5년 후 개정안 [6,8]의 개발을 가속화시켰다. 이러한 지침이 몇몇 무작위 임상시험 보고의 질을 향상시켰지만 [9,10], 여전히 많은 시험의 보고는 충분하지 않다 [2]. 뿐만 아니라 2001년 마지막 개정 이후 새로운 방법론적인 증거와 추가적인 경험이 쌓였다. 그 결과, 우리는 2001 지침을 개정하기 위한 CONSORT 그룹 회의를 조직하였다 [6-8]. 우리는 이런 과정의 결과물로, CONSORT 2010을 소개하고자 한다.

CONSORT 2010의 취지

CONSORT 2010 지침은 25개 점검항목으로 구성된 표 (Table 1)와 흐름도 (Figure 1)가 포함된 이 글이다. 이는 모든 무작위 임상시험을 보고하기 위한 지침을 제공하나, 가장 보편적인 설계형태--개인별 무작위배정, 두 그룹, 평행 임상시험에 초점을 맞추고 있다. 집단 무작위 임상시험과 비열등성 임상시험과 같은 다른 시험 설계는 다양한 추가적인 정보를 필요로 한다. 이러한 설계에 관한 CONSORT 확장 [11,12] 및 다른 결과물은 CONSORT 웹사이트 (http://www.consort-statement.org)를 통해 찾아볼 수 있다. CONSORT 지침과 더불어, 각 점검항목이 포함된 이유와 방법론적 배경을 설명하고, 앞서 발표된 투명한 보고의 예를 보여주는 상세 설명 및 기술 [13]을 개정하였다.

저자들이 점검항목을 준수하면, 명백하고, 완전하며, 투명한 보고를 가능하게 한다. 애매하거나 누락된 사항이 없는 명백한 기술은 모든 독자들에게 가장 이익이 되는 기술이다. CONSORT 2010 지침은 시험을 설계하고 수행하고 분석하기 위한 권고사항은 포함하고 있지 않다. 이 지침은 오로지 무엇이 행해졌으며 무엇이 발견되었는지에 대한 보고만을 다룬다.

그럼에도 불구하고, CONSORT는 간접적으로 설계와 수행에 영향을 미친다. 투명한 보고는, 만약 시험에서의 결점이 존재한다면 이를 드러낸다. 따라서, 부적합한 시험을 수행하였지만 투명한 보고를 해야만 하는 연구수행자는, 시험의 불충분함을 밝히지 않고서는 출판과정을 통과할 수 없게 될 것이다. 최근에 대두되고 있는 이러한 현실은 우리의 이차적이며 간접적인 목적인, 미래의 시험 설계와 수행을 향상시키고자 하는 추진력을 제공할 것이다. 게다가, CONSORT는 연구자들의 연구 설계에 도움이 될 수 있다.

CONSORT의 배경

무작위 임상시험의 보고를 발전시키고자 하는 노력은 1990년 중반 가속화되었는데, 방법론 연구도 부분적으로 이러한 가속화의 원인이 되었다. 수년간 여러 연구자들은, 이런 시험들이 제대로 보고되고 있지 않다는 것을 입증하였다. 또한, 일부 제대로 수행되지 않거나 제대로 보고되지 않는 시험들은 편향된 결과와 관련되어 있다는 경험적 근거가 쌓이기 시작했다 [14]. 보고 지침 개발을 목표로 하는 두 시도는, 우리 중 한 명(D.M.)과 Drummond Rennie를 통해 1996년 첫 번째 CONSORT 지침을 조직함으로써 결실을 맺게 되었다 [5]. 뒤따른 유사한 주제에 대한 방법론 연구는 이전의 발견을 보강하여 [15] 2001년 개정의 밑거름이 되었다 [6-8]. 그 후에 이어서, 방법론 연구가 활발해지면서 CONSORT 2010을 정교하게 하는데 영향을 미쳤다. CONSORT 데이터베이스는 700개 이상의 연구들로 구성되어 있는데(CONSORT 웹사이트에 위치), 이 데이터베이스는 CONSORT의 목적에 토대가 되는 경험적인 근거를 제공한다.

게다가, CONSORT 그룹 구성원들은 끊임없이 문헌을 추적 관찰한다. 이러한 노력으로 모인 정보들은 CONSORT 지침 개정에 대한 증거로 쓰여진다. 이러한 증거와 임상시험자, 통계학자, 역학자, 의학 편집자들로 이루어진 폭넓은 국제적인 모임인 CONSORT 그룹의 권고를 바탕으로 항목을 추가하거나, 삭제하거나 수정한다. CONSORT 위원(K.F.S., D.G.A., D.M.)은 이미 자리잡은 연구자와 떠오르는 연구자의 균형을 꾀하기 위해 노력했다. 그룹의 구성원은 매우 역동적이다. 우리의 작업이 새로이 떠오르는 과제와 필요한 전문분야에 대응하여 확장됨에 따라, 우리는 이에 기여할 새로운 구성원을 맞아들였다. 그러함으로, CONSORT는 새로운 생각과 관점을 끊임없이 흡수한다. 이러한 과정은 CONSORT 지침이 지속적으로 진화하는데 반영되었다.

시간이 지나면서, CONSORT는 많은 지지를 받았다. 전세계에 걸쳐 다양한 언어로 출판된 400편이 넘는 학술지가 CONSORT 지침을 명백히 지지하고 있다. 우리도 모르게 여러 다른 의료학술지도 지지하고 있다. 게다가, 수천 편에 이르는 추가적인 학술지는 International Committee of Medical Journal Editors (http://www.icmje.org)를 통해 CONSORT 지침을 승인함으로 함축적으로 이를 지지하고 있다. 다른 저명한 편집 모임인, Council of Science Editors 와 World Association of Medical Editors에서도 공식적으로 CONSORT를 지지한다. 이러한 지지는 정당한 것으로 생각되는데, 저자와 학술지에 의해 CONSORT가 사용될 때 보고 내용의 질이 향상되는 것으로 여겨지기 때문이다 [9].

CONSORT 2010의 개발

CONSORT 2010 그룹의 구성원 31명은, CONSORT 2001 지침을 개정하기 위해 2007년 1월 캐나다 몽트벨로에 모였다. 2001년 이후, 기존 존재하는 점검항목과 연관되어 축적된 증거뿐만 아니라 여러 새로운 주제들이 부각되었다. 몇몇 참가자들에게는, 관심되는 특정 점검항목과 관련된 증거를 종합하고 통합하는 주된 책임이 주어졌다. 이러한 증거를 기반으로, 그룹은 각 항목의 중요성에 대해 심사 숙고하였다. 이전의 CONSORT 버전에서와 같이, 우리는 무작위 임상시험 보고에 절대적으로 핵심이라 간주되는 항목들만 유지하였다. 게다가, 기관위원회의 승인과 같은 항목처럼, 시험에는 핵심적일 수 있는 항목이라도 제외된 항목이 있다. 왜냐하면, 재정단체들이 엄격하게 윤리적 검토를 강화하고 있으며, 의학학술지들은 대개 저자를 위한 설명문에 기관위원회에 의한 검토에 대한 보고를 하도록 언급하고 있기 때문이다. 예를 들어 현장 실사가 행해졌는지를 보고하는 것과 같은 항목은 바람직할 수는 있으나, 이에 대한 경험적 증거 또는 가치에 대한 합의가 결여되어 현 시점에서 이를 포함시킬지에 대한 우려가 있다. 그러므로 CONSORT 2010 지침은 최소한의 기준을 논하고자 하나, 저자들이 중요하다고 생각하는 다른 정보가 있다면 이를 포함하는 것을 막아서는 안 될 것이다.

모임 후, CONSORT 위원들은 점검표를 개정하기 위한 전화 및 대면 회의를 소집하였다. 일곱 번의 주요 수정을 반복한 후, 개정된 점검표는 피드백을 위해 구성원 전체에게 배포되었다. 피드백을 받은 후, 위원들은 두 번의 대면 모임을 통해 모든 조언을 고려하고 최종 직전 버전을 만들었다. 이는 이 글의 초안의 근간이 되었고, 후에 피드백을 받고자 전체 구성원에게 배포되었다. 그들의 조언들을 고려한 후, 위원들은 지침을 마무리하였다.

그 후 CONSORT 위원들은 다른 구성원의 도움을 받아 개정된 상세 설명 및 기술의 초안을 작성하였다. 2007 CONSORT 모임의 내용을 이용하여 개정이 진행되었다. 개정된 상세 설명 및 기술에 대한 원고는 추가, 삭제, 교정을 위해 전체 구성원에게 배포되었다. 이러한 최종적인 반복 과정은 CONSORT 2010 상세 설명 및 기술로 귀결되었다 [13].

CONSORT 2010의 변화

수정과정은 점검표의 혁신적이기보다 점진적인 변화로 결실을 맺었고 (Table 1), 흐름도 역시 단어 하나만 제외하면 바뀌지 않았다 (Figure 1). 또한, 점검표와 관련된 보고 지침 중에는 항목을 번호로 지칭하는 것들이 있기 때문에, 2번과 5번의 사이 몇몇 항목 재배치를 제외하고는 현재의 항목번호를 유지하였다. 추가되는 항목은 현존 항목 아래의 종속항목으로나, 점검표 끝의 새로운 항목번호로나, 또는(항목 3에서) 재번호화된 부분의 첨언된 항목으로 삽입하였다. 우리는 주목할 만한 전반적인 변화를 부록 1에, 구체적 변화를 부록 2에 정리하였다. CONSORT 웹사이트에서 2001과 2010 버전을 나란히 비교한 것을 볼 수 있다.

영향 및 제한

우리는, 저자들이 무작위 임상시험 보고를 작성하는 것을 돕기 위해, 편집자와 검토자가 출판을 위한 원고를 검토할 수 있게, 독자들이 출판된 글을 비판적으로 평가하도록, CONSORT 2010을 개발하였다. CONSORT 2010 상세 설명 및 기술은 점검항목에 대한 명쾌한 설명과 전후 관계 맥락을 제공한다. 시험을 완전하고 명료하고 투명하게 보고하기 위하여, 그리고 출판된 시험 보고의 평가를 돕기 위해서, 점검표와 더불어 상세 설명과 기술을 사용할 것을 강력히 권고한다.

CONSORT 2010은 문헌상 시험의 절반 이상을 차지하는 두 그룹 평행 설계 무작위 임상시험에 주된 초점을 맞추고 있다 [2]. 그러나, CONSORT 2010의 대부분의 항목은 모든 무작위 임상시험과 관련되어 있다. 그럼에도 불구하고, 몇몇 시험 종류나 시험 상황에는 시험 보고에 추가적인 정보가 필요하다. 불확실한 경우, 저자, 편집자, 독자는 CONSORT 웹사이트를 참고하여 CONSORT 내용의 확장, 확대(부연), 시행, 관련된 안내가 있는지 확인해야 한다.

CONSORT를 위해 우리가 사용한 근거바탕의 접근법은, 다른 형태의 보고를 위한 지침의 개발에도 사용되었다. 즉, 개입효과를 평가하기 위한 체계적 문헌고찰과 메타분석의 보고에 대한 지침 [16]과, 진단 연구 [17], 관찰연구 [18]의 보고에 대한 지침 등의 개발을 위한 모델로 사용되었다. 이러한 모든 시도의 분명한 목적은 보고를 향상시키기 위함이다. 보건의료 연구의 질과 투명성의 강화 (Enhancing the quality and transparency of health research; EQUATOR) 체계는 보고를 위한 지침 개발을 촉진하고자 하며, 이러한 지침을 전파하고자 한다. http://www.equator-network.org에서는 보건의료 연구분야에서 보고를 위한 모든 지침에 대한 정보를 제공한다.

CONSORT 2010에서도, 우리는 무작위 임상시험 보고를 위한 엄격한 구조가 만들어지는 것을 피했다. 실은, Standards of Reporting Trials (SORT) [19]는 엄격한 구성방식을 시도하였으나 편집자와 저자들에서의 초기 시도에서 실패로 돌아갔다 [20]. 결과적으로, 글의 구성방식은 학술지의 경향, 편집의 방향, 연구 분야의 관행, 그리고 가능하다면 저자의 선호에 따라야 한다. 우리는 보고형식을 표준화하는 것을 바라지 않는다. 저자들은 충분한 상세사항과 명쾌함을 갖춰 글에서 점검항목을 언급하기만 하면 된다. 특히 방법과 결과 부분과 같은 주요 부분에서는 부제목을 자주 쓰는 것이 원고에 유익하다고 생각한다.

CONSORT는 실제 시험 설계와 수행을 그대로 반영하는 완전하고 명료하며 투명한 보고를 촉구한다. 그러나, 잠재적인 결점으로 보고 지침은 저자들로 하여금 실제로 행한 것을 보고하기보다 지침에서 제안된 정보를 가상적으로 보고하게끔 할 수 있다. 저자, 검토자, 편집자는 이러한 잠재적 결점에 주의해야 하며, 시험계획서, 시험 등록 정보, 관리당국의 웹사이트 등을 참고하여야 한다. 게다가, CONSORT 2010 지침은 무작위 임상시험의 설계와 수행에 관한 권고는 포함하고 있지 않다. 항목들은 저자들이 무엇을 어떻게 행했는지 명료하게 설명할 수 있도록 해 주어야 하나, 저자들이 시행했어야 하는 조치를 설명하지는 않는다. 이와 같이, CONSORT 2010은 시험의 질을 평가하기 위한 도구로 고안된 것이 아니다. 점검표로 "질적 점수"를 만들어 내기 위해 사용하는 것 또한 바람직하지 않다.

그래도 우리는 연구자가 시험을 시작할 때, 최종적으로 출판될 내용을 염두에 두기를 제안한다. 의도 했건 안 했건, 제대로 하지 않은 보고는 저자가 시험의 단점에 대한 비판을 받는 것을 피하게 해준다. 그러나, 학술지와 편집자들이 CONSORT를 널리 채택함으로써, 대부분의 저자는 시험의 모든 중요한 측면을 투명하게 보고해야만 한다. 이에 따르는 면밀한 검토로 인해 잘 수행된 시험은 보상을 받게 되고, 형편없이 시행된 시험은 불이익을 받게 된다. 연구자는 시험을 설계하고 수행할 때 엄격한 기준을 따름에 대한 보상으로, CONSORT 2010 지침을 시험 시작 전에 충분히 이해해야 한다.

CONSORT 2010은 2001년에 출판된 이전 버전을 대체한다. 학술지와 편집자들로부터 축적된 이전 버전에 대한 지원은, 다른 구체적인 요구가 있지 않은 한, 이 새로운 버전에 자동적으로 이어질 것이다. 현재 CONSORT를 지원하지 않는 학술지는 CONSORT 웹

사이트에 등록하는 것으로 지원을 할 수 있을 것이다. 만약 학술지가 CONSORT를 지원하거나 지지한다면, CONSORT 2010의 원래 버전 중 하나와, CONSORT 2010 상세 설명과 기술, 그리고 CONSORT 웹사이트를 학술지의 "투고 요령"에 인용해야 한다. CONSORT를 인용하고자 하는 저자들에게 이 문서 또는 다른 저널의 CONSORT 2010 지침, 그리고 필요하다면 CONSORT 2010 상세 설명과 기술 [13]을 인용할 것을 제안한다. CONSORT 자료는 출판된 학술지나 CONSORT 웹사이트를 통해 열람할 수 있다. CONSORT 2010 지침을 다른 언어로 번역하기를 원하는 단체나 개인은 CONSORT 웹사이트에서 먼저 번역방침을 참고하여야 한다.

우리는 CONSORT 2010이 진화하는 가이드라인을 대표하고 있다고 강조한다. 이는 끊임없는 재평가와, 필요한 경우, 수정을 필요로 한다. 추후에 발생하는 의견, 비평, 경험, 그리고 누적되는 새로운 근거를 고려하여 CONSORT 자료는 더 수정될 것이다. 독자들에게 CONSORT 웹사이트에 충고사항을 제안하도록 요청한다.

부록 1: 주목할만한 CONSORT 2010 지침의 전반적 변화

항목 1,8,10,13,15,16,18,19,21등의 항목에서 단어를 단순화하고 명료화시켰다.

우리는 2001 버전에 있었던 명령형 동사를 제거하여, 항목에 걸쳐 형식의 통일성을 꾀하였다. 어떤 항목은 종속항목으로 나눔으로써 평가의 구체성을 높였다. 많은 학술지들이 저자들로 하여금 원고에서 항목이 다루고 있는 위치를 표시하여 CONSORT 점검표를 작성하도록 하고 있다. 항목이 여러 요소로 구성된 경우, 점검표 작성에 실제적 어려움이 있다는 것을 깨닫게 되었다. 예를 들면, 항목 4는 참가자들의 적격기준과 자료수집의 조건과 장소를 언급하고 있다. 2001 버전을 이용한 경우, 예를 들어, 조건과 장소에 대한 보고 없이 적격기준만 보고하여도 저자가 이 항목이 포함되어 있는 원고의 페이지 번호를 점검표에 기재하는 것이 가능하였다. CONSORT 2010은 혼동을 없애고 저자들이 적격기준과 조건에 대한 페이지 번호를 모두 점검표에 기재하도록 하였다.

부록 2: 주목할만한 CONSORT 2010 지침의 구체적 변화

항목 1b (제목과 초록) – 연구의 설계, 방법, 결과, 그리고 결론에 관한 구조적인 요약을 제시하는 것에 대한 종속항목을 추가하고, 초록에 관한 CONSORT를 참조하도록 하였다 [21].
항목 2b (서론) – "구체적 목표 또는 가설"에 대한 새로운 종속항목(CONSORT 2001의 이전 항목 5)을 추가하였다.
항목 3a (시험설계) – 이 종속항목을 포함하는 새로운 항목을 추가하여, 기본 시험 설계(평행, 교차, 집단)와 배정비율을 명시하도록 하였다.
항목 3b (시험설계) – 시험개시 이후의 방법상의 중대한 변화를 원인과 함께 기술하는 새로운 종속항목을 추가하였다.
항목 4 (참가자) – CONSORT 2001의 이전 항목 3.
항목 5 (개입) – CONSORT 2001의 이전 항목 4. 개입에 대한 기술에 "재현이 가능할 정도로 충분한" 세부정보를 포함할 것을 명시하여, 보다 구체성을 강조하였다 [3].
항목 6 (결과) – 시험이 시작된 후 일차와 이차 결과의 어떠한 변화라도 밝히도록 종속항목을 추가하였다. 저자들이 흔히 연구계획서에는 미리 명시되지 않은 일차, 이차 결과에 관한 분석을 보고하는 한편, 미리 명시된 결과 분석은 하지 않는 경우가 흔하다는(즉 선택적 결과보고를 한다는) 경험적 증거로부터 비롯되었다 [4,22]. 측정 질을 강화하기 위한 방법적인 부분에 관한 모든 문구는 제외하였다.
항목 9 (은폐방법) – 부정확하고 진부한 은폐방법에 대한 장담보다 저자들이 은폐를 위해 실제 시행된 조치를 보고하는 것을 강화하기 위하여, 은폐방법에서의 보고 주제와 설명자를 은폐방법에 포함하도록 말을 바꿨다.
항목 11 (눈가림) – 어떻게 눈가림이 행해졌는지에 대한 구체적 설명과, 적절하다면 개입과 절차의 유사성에 대한 자세한 서술을 추가하였다. 또한 눈가림(감춤)의 성공을 어떻게 평가할지에 관한 문구를 제외하였다. 이는 실제적으로 이의 수행을 지원하는 경험적 지식의 결여와 더불어 이러한 평가절차의 타당성에 대한 이론적인 우려 때문이다 [23,24].
항목 12a (통계적 방법) – 이차 결과 분석에 대한 통계적 방법 역시 제공되도록 추가하였다.
세부항목 14b (모집) – 경험적 연구를 바탕으로, "왜 시험이 끝나거나 중단되었는지"에 대한 종속항목을 추가하였다 [25].
항목 15 (시작시점 자료) – 표로 각 그룹의 기초적 특성과 임상적 특성이 가장 명료히 표현되도록 "표"가 필요함을 명시하였다.
항목 16 (분석된 수) – 흔하게 잘못 쓰는 표현인 "의도한 치료에 따른" 분석에 대해 언급하는 것을 대신하여, 원래 배정된 그룹에 참가자들을 유지하는 것에 관한 정보를 명확하게 요청하도록 하였다 [26].
세부항목 17b (결과와 추정) – 적절한 임상적 해석을 위해, 유력한 경험을 바탕으로 "이분형 자료는 절대적인 효과크기와 상대적인 효과크기를 밝히는 것을 권고한다"라는 말을 추가하도록 하였다 [27].
항목 19 (위해) – 위해와 관련된 CONSORT에 대한 참조를 포함하였다 [28].
항목 20 (한계) – "해석"이라는 주제를 변경하고, 이전 문구를 잠재적 치우침과 비정밀도의 원인을 보고하는 데 초점을 맞춘 문구로 대체하였다.
항목 22 (해석) – "전반적 증거"라는 주제를 변경하였다. 물론, 저자들이 이런 모호한 주제에서는 해석에 대한 재량을 허용 받아야 한다는 것은 알고 있다. 그렇지만, CONSORT 그룹에서는 논문의 결론 중에는 실제의 분석 결과를 잘못 표현하거나, 위해를 언급하지 않거나 실제보다 축소하여 표현하는 결론들이 있다는 것에 대해 우려를 표시하였다. 그러므로 점검항목을 수정하여, 연구결과가 해석과 일치하는, 편익과 위해가 균형을 유지하는 개념을 포함하도록 하였다.
항목 23 (등록) – 시험 등록에 대한 새 항목을 추가하였다. 경험적 증거들이 시험 등록에 대한 필요를 제기하고 있으며, 최근에는 학술지 편집자들도 시험 등록을 요구하는 추세이다 [29].
항목 24 (연구계획서) – 연구 계획서의 공개 여부(availability)에 대한 새로운 항목을 추가하였다. 저자들은 때로 시험의 수행과 보고에서 있어 연구계획서에 기술했던 내용을 무시한다는 경험적 증거들이 있다 [4,22]. 이런 이유로, 연구계획서의 공개 여부는 출판 전에는 연구계획서를 고수하도록 할 수 있으며, 출판 후에는 연구계획서를 준수한 여부를 평가하는 것을 쉽게 할 수 있다.
항목 25 (연구비) – 연구비에 대한 새로운 항목을 추가하였다. 추정된 치료효과가 연구비와 연관되어 있음을 경험적 증거들이 보여주고 있다 [30].

REFERENCES

1. Juni P, Altman DG, Egger M. Systematic reviews in health care: Assessing the quality of controlled clinical trials. BMJ 2001;323:42-46.
2. Chan AW, Altman DG. Epidemiology and reporting of randomised trials published in PubMed journals. Lancet 2005;365:1159-1162.
3. Glasziou P, Meats E, Heneghan C, Shepperd S. What is missing from descriptions of treatment in trials and reviews? BMJ 2008;336:1472-1474.

4. Dwan K, Altman DG, Arnaiz JA, Bloom J, Chan AW, Cronin E, et al. Systematic review of the empirical evidence of study publication bias and outcome reporting bias. PLoS One 2008;3:e3081.
5. Begg C, Cho M, Eastwood S, Horton R, Moher D, Olkin I, et al. Improving the quality of reporting of randomized controlled trials. The CONSORT statement. JAMA 1996;276:637-639.
6. Moher D, Schulz KF, Altman DG. The CONSORT statement: revised recommendations for improving the quality of reports of parallel-group randomised trials. Lancet 2001;357:1191-1194.
7. Moher D, Schulz KF, Altman DG, Consort G. The CONSORT statement: revised recommendations for improving the quality of reports of parallel-group randomized trials. Ann Intern Med 2001;134:657-662.
8. Moher D, Schulz KF, Altman D, Group C. The CONSORT statement: revised recommendations for improving the quality of reports of parallel-group randomized trials. JAMA 2001;285:1987-1991.
9. Plint AC, Moher D, Morrison A, Schulz K, Altman DG, Hill C, et al. Does the CONSORT checklist improve the quality of reports of randomised controlled trials? A systematic review. Med J Aust 2006;185:263-267.
10. Hopewell S, Dutton S, Yu LM, Chan AW, Altman DG. The quality of reports of randomised trials in 2000 and 2006: comparative study of articles indexed in PubMed. BMJ 2010;340:c723.
11. Campbell MK, Elbourne DR, Altman DG, group C. CONSORT statement: extension to cluster randomised trials. BMJ 2004;328:702-708.
12. Piaggio G, Elbourne DR, Altman DG, Pocock SJ, Evans SJ, Group C. Reporting of noninferiority and equivalence randomized trials: an extension of the CONSORT statement. JAMA 2006;295:1152-1160.
13. Moher D, Hopewell S, Schulz KF, Montori V, Gotzsche PC, Devereaux PJ, et al. CONSORT 2010 explanation and elaboration: updated guidelines for reporting parallel group randomised trials. BMJ 2010;340:c869.
14. Schulz KF, Chalmers I, Hayes RJ, Altman DG. Empirical evidence of bias. Dimensions of methodological quality associated with estimates of treatment effects in controlled trials. JAMA 1995;273:408-412.
15. Moher D, Pham B, Jones A, Cook DJ, Jadad AR, Moher M, et al. Does quality of reports of randomised trials affect estimates of intervention efficacy reported in meta-analyses? Lancet 1998;352:609-613.
16. Moher D, Liberati A, Tetzlaff J, Altman DG, Group P. Preferred reporting items for systematic reviews and meta-analyses: the PRISMA statement. Ann Intern Med 2009;151:264-269, W64.
17. Bossuyt PM, Reitsma JB, Bruns DE, Gatsonis CA, Glasziou PP, Irwig LM, et al. Towards complete and accurate reporting of studies of diagnostic accuracy: The STARD Initiative. Ann Intern Med 2003;138:40-44.
18. von Elm E, Altman DG, Egger M, Pocock SJ, Gotzsche PC, Vandenbroucke JP, et al. The Strengthening the Reporting of Observational Studies in Epidemiology (STROBE) statement: guidelines for reporting observational studies. Ann Intern Med 2007;147:573-577.
19. The Standards of Reporting Trials Group. A proposal for structured reporting of randomized controlled trials. The Standards of Reporting Trials Group. JAMA 1994;272:1926-1931.
20. Rennie D. Reporting randomized controlled trials. An experiment and a call for responses from readers. JAMA 1995;273:1054-1055.
21. Hopewell S, Clarke M, Moher D, Wager E, Middleton P, Altman DG, et al. CONSORT for reporting randomised trials in journal and conference abstracts. Lancet 2008;371:281-283.
22. Chan AW, Hrobjartsson A, Haahr MT, Gotzsche PC, Altman DG. Empirical evidence for selective reporting of outcomes in randomized trials: comparison of protocols to published articles. JAMA 2004;291:2457-2465.
23. Sackett DL. Commentary: Measuring the success of blinding in RCTs: don't, must, can't or needn't? Int J Epidemiol 2007;36:664-665.
24. Schulz KF, Grimes DA. Blinding in randomised trials: hiding who got what. Lancet 2002;359:696-700.
25. Montori VM, Devereaux PJ, Adhikari NK, Burns KE, Eggert CH, Briel M, et al. Randomized trials stopped early for benefit: a systematic review. JAMA 2005;294:2203-9.
26. Hollis S, Campbell F. What is meant by intention to treat analysis? Survey of published randomised controlled trials. BMJ 1999;319:670-674.
27. Nuovo J, Melnikow J, Chang D. Reporting number needed to treat and absolute risk reduction in randomized controlled trials. JAMA 2002;287: 2813-2814.
28. Ioannidis JP, Evans SJ, Gotzsche PC, O'Neill RT, Altman DG, Schulz K, et al. Better reporting of harms in randomized trials: an extension of the CONSORT statement. Ann Intern Med 2004;141:781-788.
29. De Angelis C, Drazen JM, Frizelle FA, Haug C, Hoey J, Horton R, et al. Clinical trial registration: a statement from the International Committee of Medical Journal Editors. Ann Intern Med 2004;141:477-478.
30. Lexchin J, Bero LA, Djulbegovic B, Clark O. Pharmaceutical industry sponsorship and research outcome and quality: systematic review. BMJ 2003;326:1167-1670.
31. Hopewell S, Clarke M, Moher D, Wager E, Middleton P, Altman DG, et al. CONSORT for reporting randomized controlled trials in journal and conference abstracts: explanation and elaboration. PLoS Med 2008;5:e20.
32. Boutron I, Moher D, Altman DG, Schulz KF, Ravaud P, Group C. Extending the CONSORT statement to randomized trials of nonpharmacologic treatment: explanation and elaboration. Ann Intern Med 2008;148:295-309.
33. Gagnier JJ, Boon H, Rochon P, Moher D, Barnes J, Bombardier C, et al. Reporting randomized, controlled trials of herbal interventions: an elaborated CONSORT statement. Ann Intern Med 2006;144:364-367.
34. Zwarenstein M, Treweek S, Gagnier JJ, Altman DG, Tunis S, Haynes B, et al. Improving the reporting of pragmatic trials: an extension of the CONSORT statement. BMJ 2008;337:a2390.

Supplementary Table 1. 무작위 임상시험을 보고할 때 포함해야 할 CONSORT 2010 점검표*

대주제/소주제	항목 번호	점검항목	관련페이지
제목과 초록			
	1a	제목에 무작위 임상시험임을 밝힘	
	1b	시험설계, 방법, 결과, 결론을 구조적으로 요약(구체적인 지침을 위해, 초록을 위한 CONSORT 참고 [21,31])	
서론			
배경과 목적	2a	과학적 배경과 근거에 대한 설명	
	2b	구체적 목적 또는 가설	
방법			
시험설계	3a	배정비율을 포함한 시험설계 서술(예: 평행설계, 요인설계)	
	3b	시험 개시 이후의 방법의 중대한 변화를 원인과 함께 기술(예: 적격기준)	
참가자	4a	참가자의 적격기준	
	4b	자료가 수집된 조건과 장소	
개입	5	각 군에 대한 개입에 대한 재현이 가능하도록 세부사항을 기술, 언제 그리고 어떻게 개입이 실제적으로 시행되었는지를 포함	
결과	6a	미리 지정되고 완전히 정의된 일차결과와 이차결과, 어떻게 그리고 언제 평가되는지를 포함	
	6b	시험이 개시된 후의 시험 결과의 변경을 원인과 함께 기술	
대상수	7a	어떻게 대상수가 결정되었는지	
	7b	필요하다면, 중간평가나 중단기준에 대한 설명	
무작위배정			
서열 생성	8a	무작위배정서열을 생성하기 위해 쓴 방법	
	8b	무작위배정방법의 종류; 제한사항을 구체적으로 기술(예를 들어 블록 여부와 블록 크기)	
은폐방법	9	무작위배정서열을 시행하기 위해 쓴 방법(예를 들어 연속적으로 숫자가 기입된 봉투), 개입에 배정되기까지 서열을 은폐하기 위해 시도한 조치를 기술함	
시행	10	무작위배정서열을 생성하는 주체, 참가자를 등록하는 주체, 그리고 참가자를 개입에 배정하는 주체	
눈가림	11a	만약 눈가림이 행해졌다면, 개입에 배정 후 누구에게 눈가림이 유지되었으며(예: 참가자, 의료 관계자, 결과 분석자) 그리고 어떻게	
	11b	필요하다면, 개입의 유사성을 기술	
통계적 방법	12a	일차 결과와 이차 결과를 위해 군들을 비교하기 위한 통계적 방법	
	12b	하위군 분석, 보정 분석과 같이 추가적 분석을 위한 방법	
연구결과			
참가 흐름도 (그림을 그릴 것을 강하게 권고)	13a	각 군당, 무작위배정된 참가자 수, 의도한 치료를 받은 참가자 수, 일차 결과를 위해 분석된 참가자 수	
	13b	각 군당, 무작위배정 후 탈락과 제외를 원인과 함께 기술	
모집	14a	모집기간과 추적관찰 기간의 시작날짜와 종료 날짜	
	14b	시험이 종료되거나 중단된 이유	
시작시점 자료	15	각 군의 시작시점에서의 인구학적, 임상적 특성을 보여주는 표	
분석된 수	16	각 군당, 각 분석에 포함된 참가자 수를 기술하고 처음 배정된 군에 따라 분석되었는지 여부를 기술	
결과와 추정	17a	각 일차 결과와 이차 결과에 대해, 군당 연구결과와 추정된 유효크기와 정밀도(예를 들어 95% 신뢰구간)를 기술	
	17b	이분형 결과변수의 경우, 절대적인 유효크기와 상대적인 유효크기를 표현할 것을 권고	
부차적 분석	18	하위군 분석과 보정 분석을 포함한 추가적 분석에 대한 연구결과를 기술하고 사전에 미리 지정된 분석인지 탐색적 분석인지 구분할 것	
위해	19	군별로 모든 중대한 위해나 의도치 않은 부작용을 기술(구체적인 지침을 위해, 위해를 위한 CONSORT 참고 [28])	

Continued to the next page

Supplementary Table 1. Continued

대주제/소주제	항목 번호	점검항목	관련페이지
고찰			
한계	20	시험의 한계, 잠재적인 바이어스나, 비정밀도, 필요하다면 다중분석의 문제	______
일반화	21	시험의 발견이 일반화 가능한지(외적 타당성, 응용가능성)	______
해석	22	결과와 일치하고, 편익과 위해가 균형을 유지하며, 관련 있는 다른 증거들을 고려하고 있는 해석인지	______
다른 정보			
등록	23	등록번호와 시험등록명	______
연구계획서	24	필요하다면, 어디서 연구계획서 전문을 확인할 수 있는지	______
연구비	25	연구비의 출처와 다른 지원(예를 들어 약제의 조달) 및 재정지원자의 역할	______

CONSORT, Consolidated Standards of Reporting Trials.
*모든 항목들의 중요한 설명을 위해 CONSORT 2010 상세 설명 및 기술 [13]을 이 CONSORT와 함께 읽기를 권고합니다. 필요하다면, 집단 무작위 임상시험 [11], 비열등성과 동등성 임상시험 [12], 비약물 치료 [32], 약초 개입 [33], 실용주의적 임상시험 [34]을 위한 CONSORT 확장을 읽기를 권고합니다. 추가적인 확장도 곧 마련될 예정입니다. 이러한 문서와 점검목록과 관련된 최신 참고문헌은 http://www.consort-statement.org를 참고하십시오.

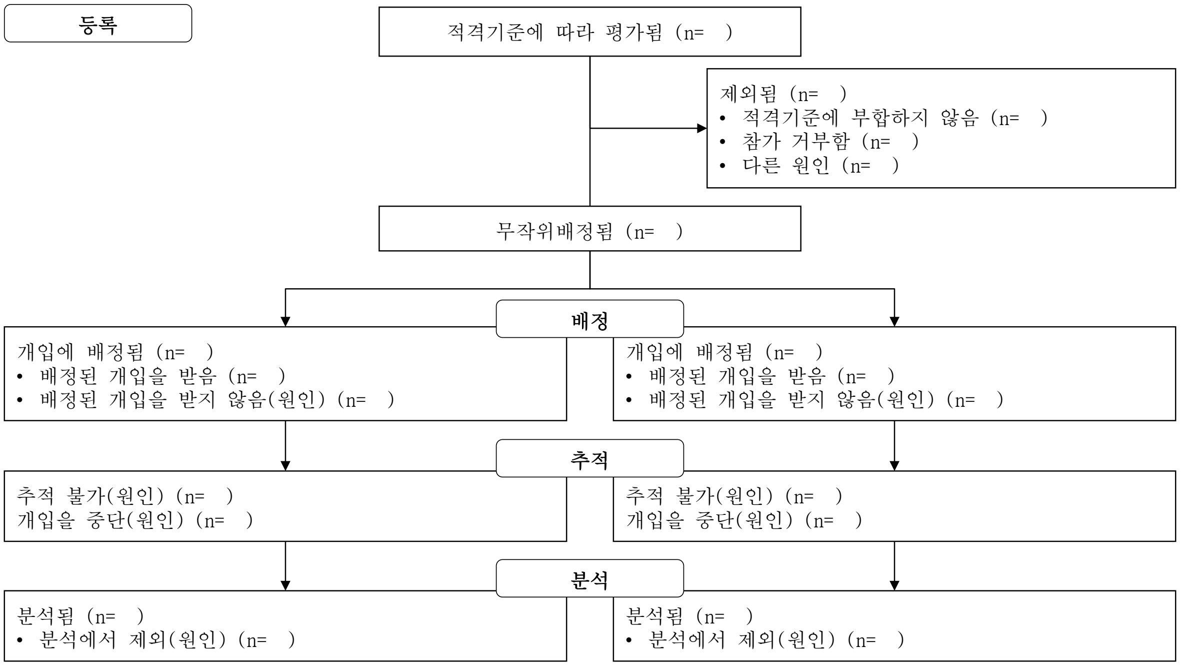

Supplementary Figure 1. 두 그룹 평행 설계 무작위 임상시험 흐름도 (등록, 배정, 추적, 분석 과정).
